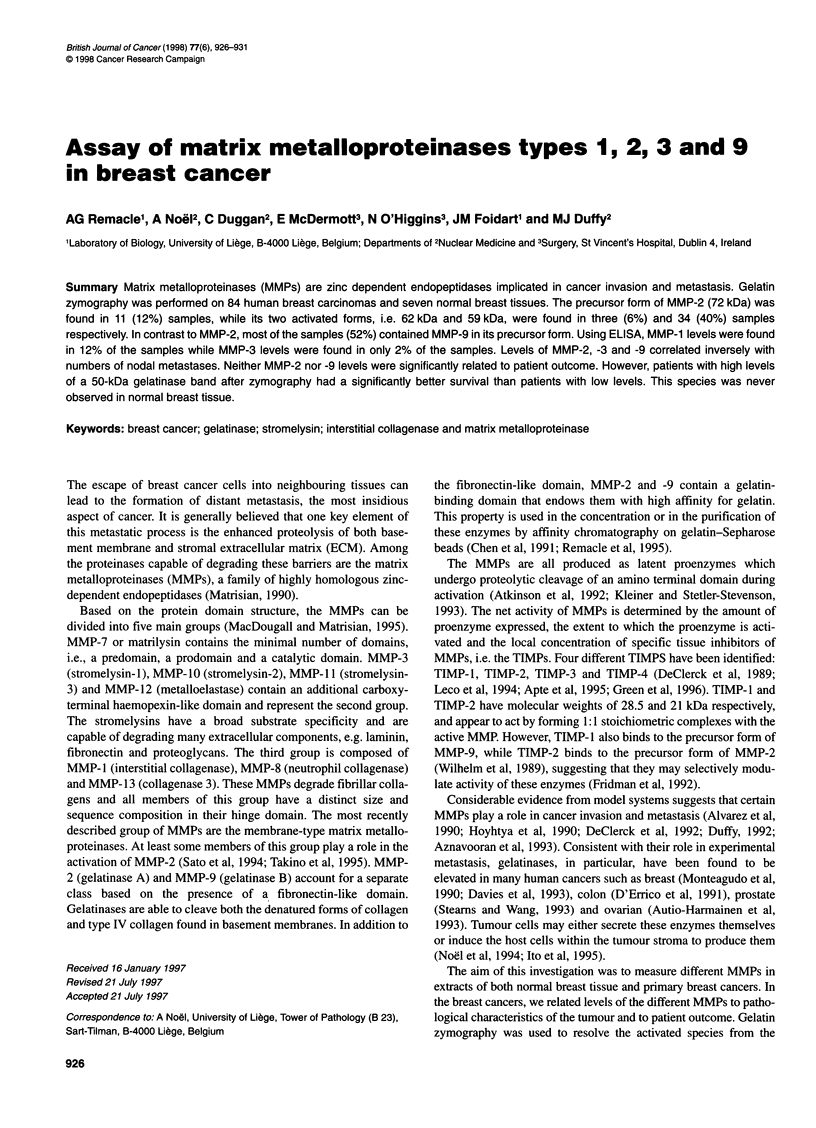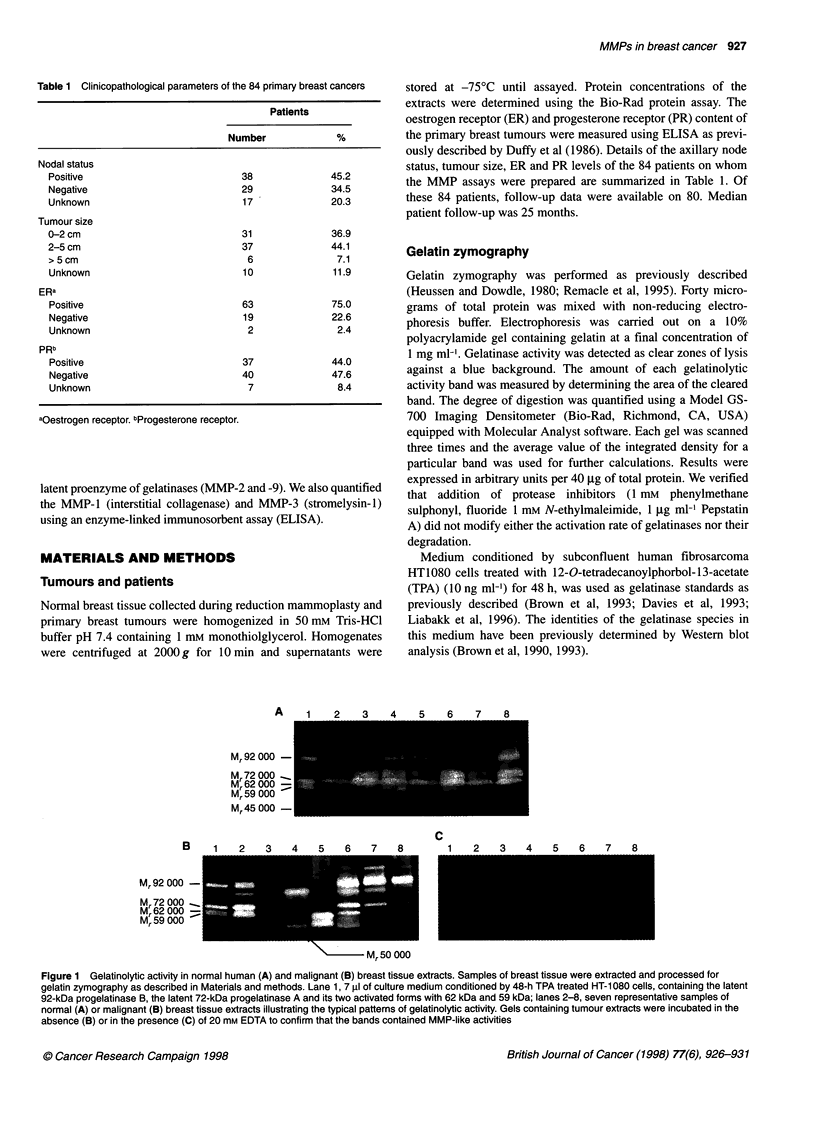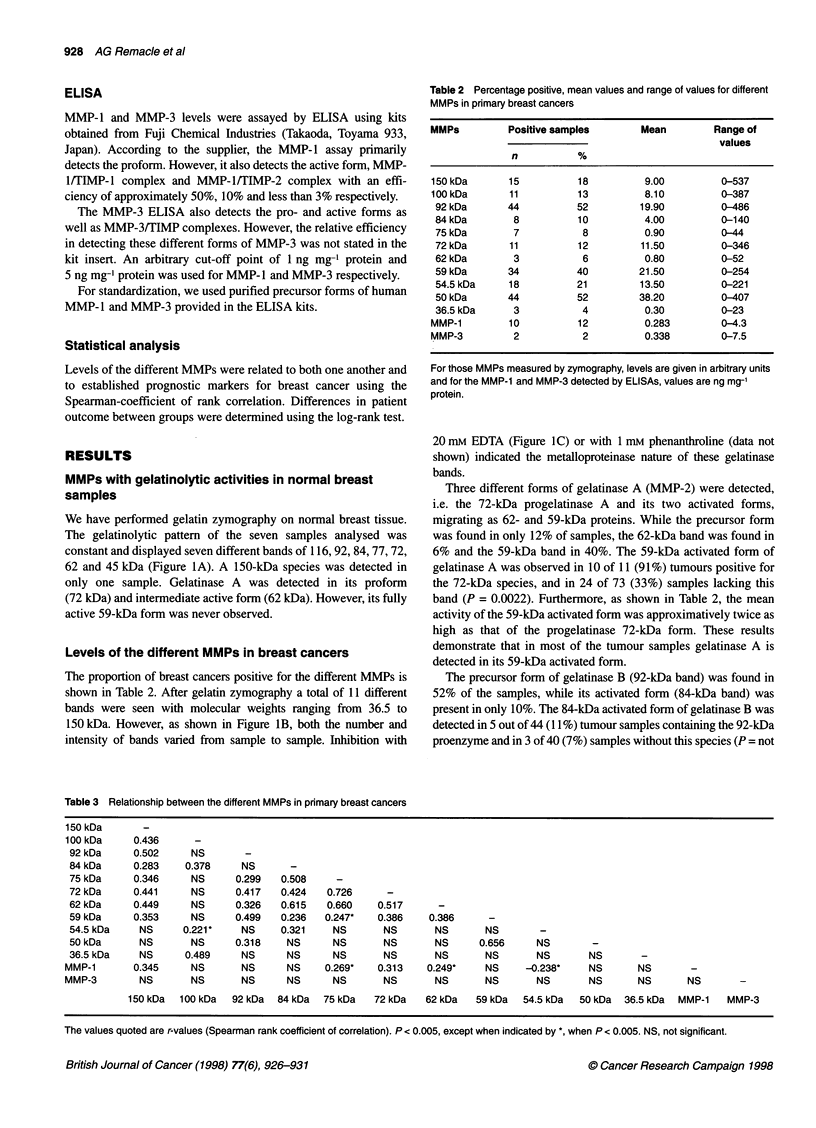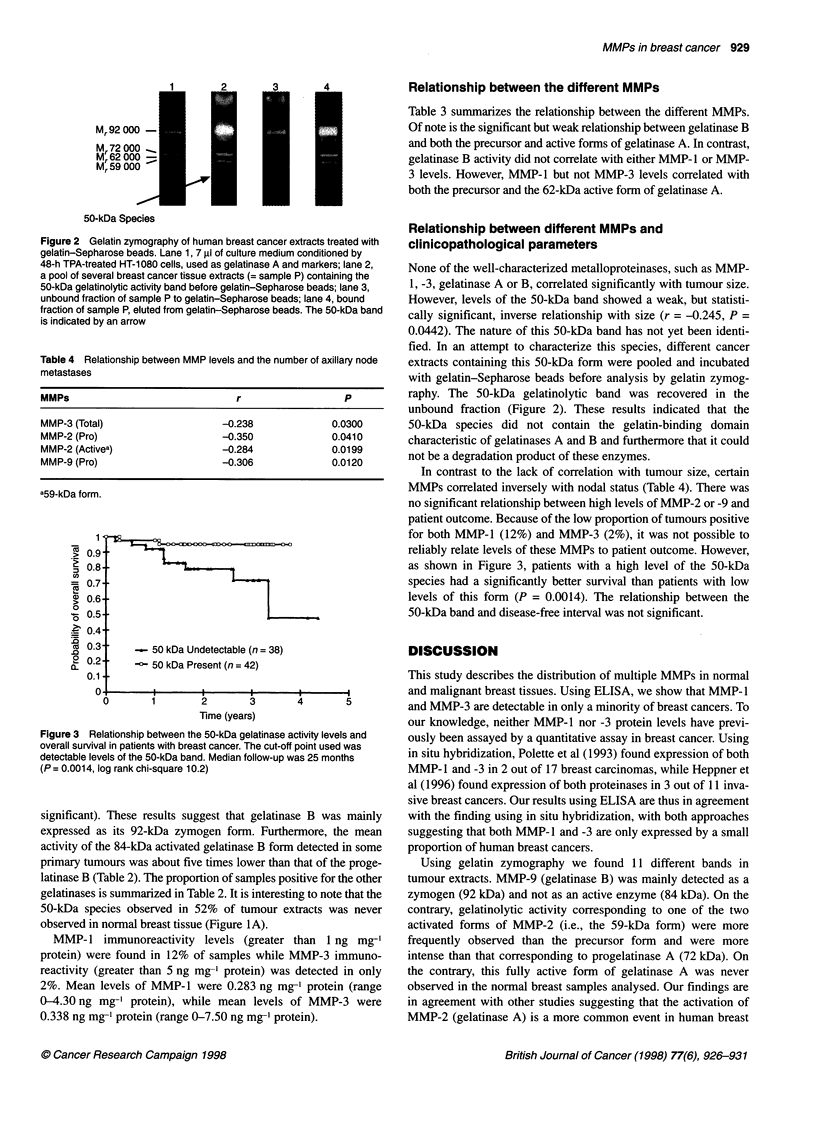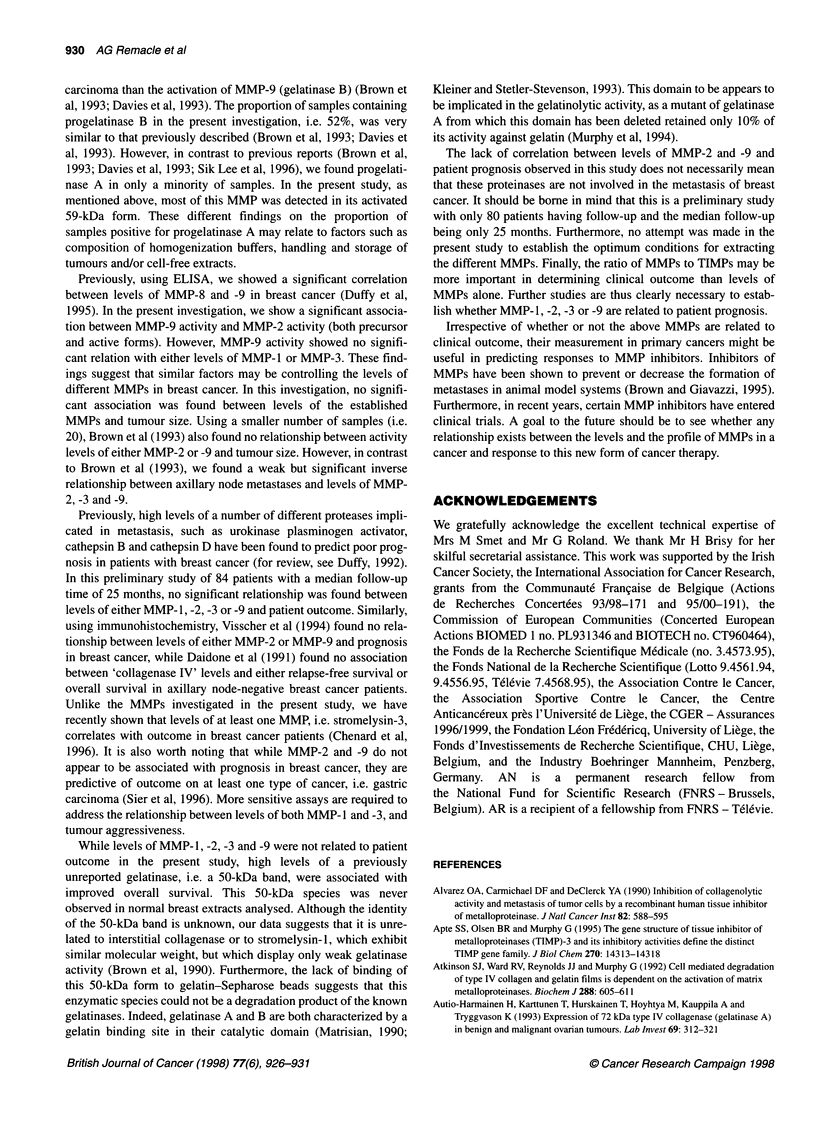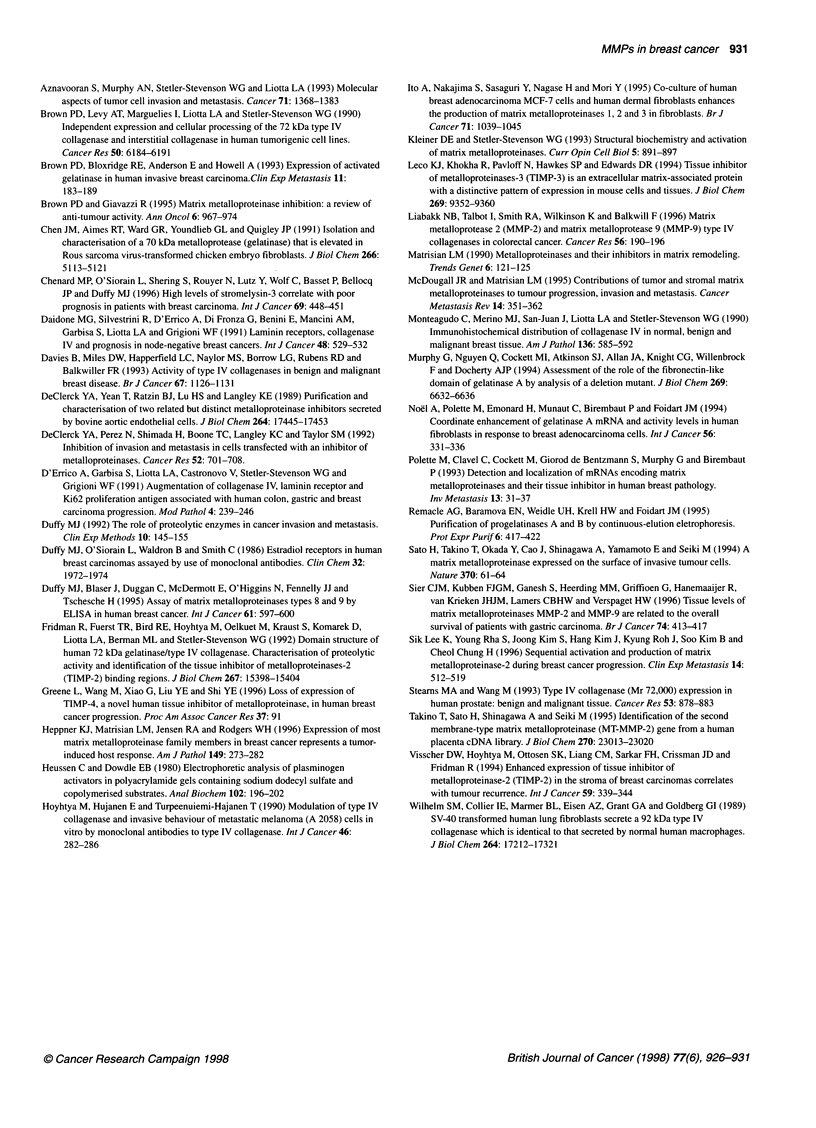# Assay of matrix metalloproteinases types 1, 2, 3 and 9 in breast cancer.

**DOI:** 10.1038/bjc.1998.153

**Published:** 1998-03

**Authors:** A. G. Remacle, A. NoÃ«l, C. Duggan, E. McDermott, N. O'Higgins, J. M. Foidart, M. J. Duffy

**Affiliations:** Laboratory of Biology, University of LiÃ¨ge, Belgium.

## Abstract

**Images:**


British Journal of Cancer (1998) 77(6), 926–931
© 1998 Cancer Research Campaign

### Assay of matrix metalloproteinases types 1, 2, 3 and 9 in breast cancer

**AG Remacle[1], A Noël[2], C Duggan[2], E McDermott[3], N O'Higgins[3], JM Foidart[1] and MJ Duffy[2]**

[1]Laboratory of Biology, University of Liège, B-4000 Liège, Belgium; Departments of [2]Nuclear Medicine and [3]Surgery, St Vincent's Hospital, Dublin 4, Ireland

**Summary** Matrix metalloproteinases (MMPs) are zinc dependent endopeptidases implicated in cancer invasion and metastasis. Gelatin zymography was performed on 84 human breast carcinomas and seven normal breast tissues. The precursor form of MMP-2 (72 kDa) was found in 11 (12%) samples, while its two activated forms, i.e. 62 kDa and 59 kDa, were found in three (6%) and 34 (40%) samples respectively. In contrast to MMP-2, most of the samples (52%) contained MMP-9 in its precursor form. Using ELISA, MMP-1 levels were found in 12% of the samples while MMP-3 levels were found in only 2% of the samples. Levels of MMP-2, -3 and -9 correlated inversely with numbers of nodal metastases. Neither MMP-2 nor -9 levels were significantly related to patient outcome. However, patients with high levels of a 50-kDa gelatinase band after zymography had a significantly better survival than patients with low levels. This species was never observed in normal breast tissue.

**Keywords:** breast cancer; gelatinase; stromelysin; interstitial collagenase and matrix metalloproteinase

The escape of breast cancer cells into neighbouring tissues can lead to the formation of distant metastasis, the most insidious aspect of cancer. It is generally believed that one key element of this metastatic process is the enhanced proteolysis of both basement membrane and stromal extracellular matrix (ECM). Among the proteinases capable of degrading these barriers are the matrix metalloproteinases (MMPs), a family of highly homologous zinc-dependent endopeptidases (Matrisian, 1990).

Based on the protein domain structure, the MMPs can be divided into five main groups (MacDougall and Matrisian, 1995). MMP-7 or matrilysin contains the minimal number of domains, i.e., a predomain, a prodomain and a catalytic domain. MMP-3 (stromelysin-1), MMP-10 (stromelysin-2), MMP-11 (stromelysin-3) and MMP-12 (metalloelastase) contain an additional carboxy-terminal haemopexin-like domain and represent the second group. The stromelysins have a broad substrate specificity and are capable of degrading many extracellular components, e.g. laminin, fibronectin and proteoglycans. The third group is composed of MMP-1 (interstitial collagenase), MMP-8 (neutrophil collagenase) and MMP-13 (collagenase 3). These MMPs degrade fibrillar collagens and all members of this group have a distinct size and sequence composition in their hinge domain. The most recently described group of MMPs are the membrane-type matrix metalloproteinases. At least some members of this group play a role in the activation of MMP-2 (Sato et al, 1994; Takino et al, 1995). MMP-2 (gelatinase A) and MMP-9 (gelatinase B) account for a separate class based on the presence of a fibronectin-like domain. Gelatinases are able to cleave both the denatured forms of collagen and type IV collagen found in basement membranes. In addition to the fibronectin-like domain, MMP-2 and -9 contain a gelatin-binding domain that endows them with high affinity for gelatin. This property is used in the concentration or in the purification of these enzymes by affinity chromatography on gelatin–Sepharose beads (Chen et al, 1991; Remacle et al, 1995).

The MMPs are all produced as latent proenzymes which undergo proteolytic cleavage of an amino terminal domain during activation (Atkinson et al, 1992; Kleiner and Stetler-Stevenson, 1993). The net activity of MMPs is determined by the amount of proenzyme expressed, the extent to which the proenzyme is activated and the local concentration of specific tissue inhibitors of MMPs, i.e. the TIMPs. Four different TIMPS have been identified: TIMP-1, TIMP-2, TIMP-3 and TIMP-4 (DeClerck et al, 1989; Leco et al, 1994; Apte et al, 1995; Green et al, 1996). TIMP-1 and TIMP-2 have molecular weights of 28.5 and 21 kDa respectively, and appear to act by forming 1:1 stoichiometric complexes with the active MMP. However, TIMP-1 also binds to the precursor form of MMP-9, while TIMP-2 binds to the precursor form of MMP-2 (Wilhelm et al, 1989), suggesting that they may selectively modulate activity of these enzymes (Fridman et al, 1992).

Considerable evidence from model systems suggests that certain MMPs play a role in cancer invasion and metastasis (Alvarez et al, 1990; Hoyhtya et al, 1990; DeClerck et al, 1992; Duffy, 1992; Aznavooran et al, 1993). Consistent with their role in experimental metastasis, gelatinases, in particular, have been found to be elevated in many human cancers such as breast (Monteagudo et al, 1990; Davies et al, 1993), colon (D'Errico et al, 1991), prostate (Stearns and Wang, 1993) and ovarian (Autio-Harmainen et al, 1993). Tumour cells may either secrete these enzymes themselves or induce the host cells within the tumour stroma to produce them (Noël et al, 1994; Ito et al, 1995).

The aim of this investigation was to measure different MMPs in extracts of both normal breast tissue and primary breast cancers. In the breast cancers, we related levels of the different MMPs to pathological characteristics of the tumour and to patient outcome. Gelatin zymography was used to resolve the activated species from the

*Received 16 January 1997*
*Revised 21 July 1997*
*Accepted 21 July 1997*

*Correspondence to:* A Noël, University of Liège, Tower of Pathology (B 23), Sart-Tilman, B-4000 Liège, Belgium

Table 1 Clinicopathological parameters of the 84 primary breast cancers

| | Patients | |
|---|---|---|
| | Number | % |
| Nodal status | | |
| Positive | 38 | 45.2 |
| Negative | 29 | 34.5 |
| Unknown | 17 | 20.3 |
| Tumour size | | |
| 0–2 cm | 31 | 36.9 |
| 2–5 cm | 37 | 44.1 |
| > 5 cm | 6 | 7.1 |
| Unknown | 10 | 11.9 |
| ER[a] | | |
| Positive | 63 | 75.0 |
| Negative | 19 | 22.6 |
| Unknown | 2 | 2.4 |
| PR[b] | | |
| Positive | 37 | 44.0 |
| Negative | 40 | 47.6 |
| Unknown | 7 | 8.4 |

[a]Oestrogen receptor. [b]Progesterone receptor.

latent proenzyme of gelatinases (MMP-2 and -9). We also quantified the MMP-1 (interstitial collagenase) and MMP-3 (stromelysin-1) using an enzyme-linked immunosorbent assay (ELISA).

### MATERIALS AND METHODS

### Tumours and patients

Normal breast tissue collected during reduction mammoplasty and primary breast tumours were homogenized in 50 mM Tris-HCl buffer pH 7.4 containing 1 mM monothiolglycerol. Homogenates were centrifuged at 2000 *g* for 10 min and supernatants were stored at –75°C until assayed. Protein concentrations of the extracts were determined using the Bio-Rad protein assay. The oestrogen receptor (ER) and progesterone receptor (PR) content of the primary breast tumours were measured using ELISA as previously described by Duffy et al (1986). Details of the axillary node status, tumour size, ER and PR levels of the 84 patients on whom the MMP assays were prepared are summarized in Table 1. Of these 84 patients, follow-up data were available on 80. Median patient follow-up was 25 months.

### Gelatin zymography

Gelatin zymography was performed as previously described (Heussen and Dowdle, 1980; Remacle et al, 1995). Forty micrograms of total protein was mixed with non-reducing electrophoresis buffer. Electrophoresis was carried out on a 10% polyacrylamide gel containing gelatin at a final concentration of 1 mg ml$^{-1}$. Gelatinase activity was detected as clear zones of lysis against a blue background. The amount of each gelatinolytic activity band was measured by determining the area of the cleared band. The degree of digestion was quantified using a Model GS-700 Imaging Densitometer (Bio-Rad, Richmond, CA, USA) equipped with Molecular Analyst software. Each gel was scanned three times and the average value of the integrated density for a particular band was used for further calculations. Results were expressed in arbitrary units per 40 μg of total protein. We verified that addition of protease inhibitors (1 mM phenylmethane sulphonyl, fluoride 1 mM *N*-ethylmaleimide, 1 μg ml$^{-1}$ Pepstatin A) did not modify either the activation rate of gelatinases nor their degradation.

Medium conditioned by subconfluent human fibrosarcoma HT1080 cells treated with 12-*O*-tetradecanoylphorbol-13-acetate (TPA) (10 ng ml$^{-1}$) for 48 h, was used as gelatinase standards as previously described (Brown et al, 1993; Davies et al, 1993; Liabakk et al, 1996). The identities of the gelatinase species in this medium have been previously determined by Western blot analysis (Brown et al, 1990, 1993).

**Figure 1** Gelatinolytic activity in normal human (**A**) and malignant (**B**) breast tissue extracts. Samples of breast tissue were extracted and processed for gelatin zymography as described in Materials and methods. Lane 1, 7 μl of culture medium conditioned by 48-h TPA treated HT-1080 cells, containing the latent 92-kDa progelatinase B, the latent 72-kDa progelatinase A and its two activated forms with 62 kDa and 59 kDa; lanes 2–8, seven representative samples of normal (**A**) or malignant (**B**) breast tissue extracts illustrating the typical patterns of gelatinolytic activity. Gels containing tumour extracts were incubated in the absence (**B**) or in the presence (**C**) of 20 mM EDTA to confirm that the bands contained MMP-like activities

© Cancer Research Campaign 1998

### ELISA

MMP-1 and MMP-3 levels were assayed by ELISA using kits obtained from Fuji Chemical Industries (Takaoda, Toyama 933, Japan). According to the supplier, the MMP-1 assay primarily detects the proform. However, it also detects the active form, MMP-1/TIMP-1 complex and MMP-1/TIMP-2 complex with an efficiency of approximately 50%, 10% and less than 3% respectively.

The MMP-3 ELISA also detects the pro- and active forms as well as MMP-3/TIMP complexes. However, the relative efficiency in detecting these different forms of MMP-3 was not stated in the kit insert. An arbitrary cut-off point of 1 ng $mg^{-1}$ protein and 5 ng $mg^{-1}$ protein was used for MMP-1 and MMP-3 respectively.

For standardization, we used purified precursor forms of human MMP-1 and MMP-3 provided in the ELISA kits.

### Statistical analysis

Levels of the different MMPs were related to both one another and to established prognostic markers for breast cancer using the Spearman-coefficient of rank correlation. Differences in patient outcome between groups were determined using the log-rank test.

### RESULTS

### MMPs with gelatinolytic activities in normal breast samples

We have performed gelatin zymography on normal breast tissue. The gelatinolytic pattern of the seven samples analysed was constant and displayed seven different bands of 116, 92, 84, 77, 72, 62 and 45 kDa (Figure 1A). A 150-kDa species was detected in only one sample. Gelatinase A was detected in its proform (72 kDa) and intermediate active form (62 kDa). However, its fully active 59-kDa form was never observed.

### Levels of the different MMPs in breast cancers

The proportion of breast cancers positive for the different MMPs is shown in Table 2. After gelatin zymography a total of 11 different bands were seen with molecular weights ranging from 36.5 to 150 kDa. However, as shown in Figure 1B, both the number and intensity of bands varied from sample to sample. Inhibition with 20 mM EDTA (Figure 1C) or with 1 mM phenanthroline (data not shown) indicated the metalloproteinase nature of these gelatinase bands.

**Table 2** Percentage positive, mean values and range of values for different MMPs in primary breast cancers

| MMPs | Positive samples | | Mean | Range of values |
|---|---|---|---|---|
| | *n* | % | | |
| 150 kDa | 15 | 18 | 9.00 | 0–537 |
| 100 kDa | 11 | 13 | 8.10 | 0–387 |
| 92 kDa | 44 | 52 | 19.90 | 0–486 |
| 84 kDa | 8 | 10 | 4.00 | 0–140 |
| 75 kDa | 7 | 8 | 0.90 | 0–44 |
| 72 kDa | 11 | 12 | 11.50 | 0–346 |
| 62 kDa | 3 | 6 | 0.80 | 0–52 |
| 59 kDa | 34 | 40 | 21.50 | 0–254 |
| 54.5 kDa | 18 | 21 | 13.50 | 0–221 |
| 50 kDa | 44 | 52 | 38.20 | 0–407 |
| 36.5 kDa | 3 | 4 | 0.30 | 0–23 |
| MMP-1 | 10 | 12 | 0.283 | 0–4.3 |
| MMP-3 | 2 | 2 | 0.338 | 0–7.5 |

For those MMPs measured by zymography, levels are given in arbitrary units and for the MMP-1 and MMP-3 detected by ELISAs, values are ng $mg^{-1}$ protein.

Three different forms of gelatinase A (MMP-2) were detected, i.e. the 72-kDa progelatinase A and its two activated forms, migrating as 62- and 59-kDa proteins. While the precursor form was found in only 12% of samples, the 62-kDa band was found in 6% and the 59-kDa band in 40%. The 59-kDa activated form of gelatinase A was observed in 10 of 11 (91%) tumours positive for the 72-kDa species, and in 24 of 73 (33%) samples lacking this band ($P$ = 0.0022). Furthermore, as shown in Table 2, the mean activity of the 59-kDa activated form was approximatively twice as high as that of the progelatinase 72-kDa form. These results demonstrate that in most of the tumour samples gelatinase A is detected in its 59-kDa activated form.

The precursor form of gelatinase B (92-kDa band) was found in 52% of the samples, while its activated form (84-kDa band) was present in only 10%. The 84-kDa activated form of gelatinase B was detected in 5 out of 44 (11%) tumour samples containing the 92-kDa proenzyme and in 3 of 40 (7%) samples without this species ($P$ = not

**Table 3** Relationship between the different MMPs in primary breast cancers

| | | | | | | | | | | | | | |
|---|---|---|---|---|---|---|---|---|---|---|---|---|---|
| 150 kDa | – | | | | | | | | | | | | |
| 100 kDa | 0.436 | – | | | | | | | | | | | |
| 92 kDa | 0.502 | NS | – | | | | | | | | | | |
| 84 kDa | 0.283 | 0.378 | NS | – | | | | | | | | | |
| 75 kDa | 0.346 | NS | 0.299 | 0.508 | – | | | | | | | | |
| 72 kDa | 0.441 | NS | 0.417 | 0.424 | 0.726 | – | | | | | | | |
| 62 kDa | 0.449 | NS | 0.326 | 0.615 | 0.660 | 0.517 | – | | | | | | |
| 59 kDa | 0.353 | NS | 0.499 | 0.236 | 0.247* | 0.386 | 0.386 | – | | | | | |
| 54.5 kDa | NS | 0.221* | NS | 0.321 | NS | NS | NS | NS | – | | | | |
| 50 kDa | NS | NS | 0.318 | NS | NS | NS | NS | 0.656 | NS | – | | | |
| 36.5 kDa | NS | 0.489 | NS | NS | NS | NS | NS | NS | NS | NS | – | | |
| MMP-1 | 0.345 | NS | NS | NS | 0.269* | 0.313 | 0.249* | NS | –0.238* | NS | NS | – | |
| MMP-3 | NS | NS | NS | NS | NS | NS | NS | NS | NS | NS | NS | NS | – |
| | 150 kDa | 100 kDa | 92 kDa | 84 kDa | 75 kDa | 72 kDa | 62 kDa | 59 kDa | 54.5 kDa | 50 kDa | 36.5 kDa | MMP-1 | MMP-3 |

The values quoted are *r*-values (Spearman rank coefficient of correlation). $P < 0.005$, except when indicated by *, when $P < 0.005$. NS, not significant.

© Cancer Research Campaign 1998

**Figure 2** Gelatin zymography of human breast cancer extracts treated with gelatin–Sepharose beads. Lane 1, 7 µl of culture medium conditioned by 48-h TPA-treated HT-1080 cells, used as gelatinase A and markers; lane 2, a pool of several breast cancer tissue extracts (= sample P) containing the 50-kDa gelatinolytic activity band before gelatin–Sepharose beads; lane 3, unbound fraction of sample P to gelatin–Sepharose beads; lane 4, bound fraction of sample P, eluted from gelatin–Sepharose beads. The 50-kDa band is indicated by an arrow

**Table 4** Relationship between MMP levels and the number of axillary node metastases

| MMPs | *r* | *P* |
|---|---|---|
| MMP-3 (Total) | −0.238 | 0.0300 |
| MMP-2 (Pro) | −0.350 | 0.0410 |
| MMP-2 (Active[a]) | −0.284 | 0.0199 |
| MMP-9 (Pro) | −0.306 | 0.0120 |

[a]59-kDa form.

**Figure 3** Relationship between the 50-kDa gelatinase activity levels and overall survival in patients with breast cancer. The cut-off point used was detectable levels of the 50-kDa band. Median follow-up was 25 months ($P$ = 0.0014, log rank chi-square 10.2)

significant). These results suggest that gelatinase B was mainly expressed as its 92-kDa zymogen form. Furthermore, the mean activity of the 84-kDa activated gelatinase B form detected in some primary tumours was about five times lower than that of the progelatinase B (Table 2). The proportion of samples positive for the other gelatinases is summarized in Table 2. It is interesting to note that the 50-kDa species observed in 52% of tumour extracts was never observed in normal breast tissue (Figure 1A).

MMP-1 immunoreactivity levels (greater than 1 ng $mg^{-1}$ protein) were found in 12% of samples while MMP-3 immunoreactivity (greater than 5 ng $mg^{-1}$ protein) was detected in only 2%. Mean levels of MMP-1 were 0.283 ng $mg^{-1}$ protein (range 0–4.30 ng $mg^{-1}$ protein), while mean levels of MMP-3 were 0.338 ng $mg^{-1}$ protein (range 0–7.50 ng $mg^{-1}$ protein).

### Relationship between the different MMPs

Table 3 summarizes the relationship between the different MMPs. Of note is the significant but weak relationship between gelatinase B and both the precursor and active forms of gelatinase A. In contrast, gelatinase B activity did not correlate with either MMP-1 or MMP-3 levels. However, MMP-1 but not MMP-3 levels correlated with both the precursor and the 62-kDa active form of gelatinase A.

### Relationship between different MMPs and clinicopathological parameters

None of the well-characterized metalloproteinases, such as MMP-1, -3, gelatinase A or B, correlated significantly with tumour size. However, levels of the 50-kDa band showed a weak, but statistically significant, inverse relationship with size ($r = -0.245$, $P$ = 0.0442). The nature of this 50-kDa band has not yet been identified. In an attempt to characterize this species, different cancer extracts containing this 50-kDa form were pooled and incubated with gelatin–Sepharose beads before analysis by gelatin zymography. The 50-kDa gelatinolytic band was recovered in the unbound fraction (Figure 2). These results indicated that the 50-kDa species did not contain the gelatin-binding domain characteristic of gelatinases A and B and furthermore that it could not be a degradation product of these enzymes.

In contrast to the lack of correlation with tumour size, certain MMPs correlated inversely with nodal status (Table 4). There was no significant relationship between high levels of MMP-2 or -9 and patient outcome. Because of the low proportion of tumours positive for both MMP-1 (12%) and MMP-3 (2%), it was not possible to reliably relate levels of these MMPs to patient outcome. However, as shown in Figure 3, patients with a high level of the 50-kDa species had a significantly better survival than patients with low levels of this form ($P$ = 0.0014). The relationship between the 50-kDa band and disease-free interval was not significant.

### DISCUSSION

This study describes the distribution of multiple MMPs in normal and malignant breast tissues. Using ELISA, we show that MMP-1 and MMP-3 are detectable in only a minority of breast cancers. To our knowledge, neither MMP-1 nor -3 protein levels have previously been assayed by a quantitative assay in breast cancer. Using in situ hybridization, Polette et al (1993) found expression of both MMP-1 and -3 in 2 out of 17 breast carcinomas, while Heppner et al (1996) found expression of both proteinases in 3 out of 11 invasive breast cancers. Our results using ELISA are thus in agreement with the finding using in situ hybridization, with both approaches suggesting that both MMP-1 and -3 are only expressed by a small proportion of human breast cancers.

Using gelatin zymography we found 11 different bands in tumour extracts. MMP-9 (gelatinase B) was mainly detected as a zymogen (92 kDa) and not as an active enzyme (84 kDa). On the contrary, gelatinolytic activity corresponding to one of the two activated forms of MMP-2 (i.e., the 59-kDa form) were more frequently observed than the precursor form and were more intense than that corresponding to progelatinase A (72 kDa). On the contrary, this fully active form of gelatinase A was never observed in the normal breast samples analysed. Our findings are in agreement with other studies suggesting that the activation of MMP-2 (gelatinase A) is a more common event in human breast

© Cancer Research Campaign 1998

carcinoma than the activation of MMP-9 (gelatinase B) (Brown et al, 1993; Davies et al, 1993). The proportion of samples containing progelatinase B in the present investigation, i.e. 52%, was very similar to that previously described (Brown et al, 1993; Davies et al, 1993). However, in contrast to previous reports (Brown et al, 1993; Davies et al, 1993; Sik Lee et al, 1996), we found progelatinase A in only a minority of samples. In the present study, as mentioned above, most of this MMP was detected in its activated 59-kDa form. These different findings on the proportion of samples positive for progelatinase A may relate to factors such as composition of homogenization buffers, handling and storage of tumours and/or cell-free extracts.

Previously, using ELISA, we showed a significant correlation between levels of MMP-8 and -9 in breast cancer (Duffy et al, 1995). In the present investigation, we show a significant association between MMP-9 activity and MMP-2 activity (both precursor and active forms). However, MMP-9 activity showed no significant relation with either levels of MMP-1 or MMP-3. These findings suggest that similar factors may be controlling the levels of different MMPs in breast cancer. In this investigation, no significant association was found between levels of the established MMPs and tumour size. Using a smaller number of samples (i.e. 20), Brown et al (1993) also found no relationship between activity levels of either MMP-2 or -9 and tumour size. However, in contrast to Brown et al (1993), we found a weak but significant inverse relationship between axillary node metastases and levels of MMP-2, -3 and -9.

Previously, high levels of a number of different proteases implicated in metastasis, such as urokinase plasminogen activator, cathepsin B and cathepsin D have been found to predict poor prognosis in patients with breast cancer (for review, see Duffy, 1992). In this preliminary study of 84 patients with a median follow-up time of 25 months, no significant relationship was found between levels of either MMP-1, -2, -3 or -9 and patient outcome. Similarly, using immunohistochemistry, Visscher et al (1994) found no relationship between levels of either MMP-2 or MMP-9 and prognosis in breast cancer, while Daidone et al (1991) found no association between 'collagenase IV' levels and either relapse-free survival or overall survival in axillary node-negative breast cancer patients. Unlike the MMPs investigated in the present study, we have recently shown that levels of at least one MMP, i.e. stromelysin-3, correlates with outcome in breast cancer patients (Chenard et al, 1996). It is also worth noting that while MMP-2 and -9 do not appear to be associated with prognosis in breast cancer, they are predictive of outcome on at least one type of cancer, i.e. gastric carcinoma (Sier et al, 1996). More sensitive assays are required to address the relationship between levels of both MMP-1 and -3, and tumour aggressiveness.

While levels of MMP-1, -2, -3 and -9 were not related to patient outcome in the present study, high levels of a previously unreported gelatinase, i.e. a 50-kDa band, were associated with improved overall survival. This 50-kDa species was never observed in normal breast extracts analysed. Although the identity of the 50-kDa band is unknown, our data suggests that it is unrelated to interstitial collagenase or to stromelysin-1, which exhibit similar molecular weight, but which display only weak gelatinase activity (Brown et al, 1990). Furthermore, the lack of binding of this 50-kDa form to gelatin–Sepharose beads suggests that this enzymatic species could not be a degradation product of the known gelatinases. Indeed, gelatinase A and B are both characterized by a gelatin binding site in their catalytic domain (Matrisian, 1990; Kleiner and Stetler-Stevenson, 1993). This domain to be appears to be implicated in the gelatinolytic activity, as a mutant of gelatinase A from which this domain has been deleted retained only 10% of its activity against gelatin (Murphy et al, 1994).

The lack of correlation between levels of MMP-2 and -9 and patient prognosis observed in this study does not necessarily mean that these proteinases are not involved in the metastasis of breast cancer. It should be borne in mind that this is a preliminary study with only 80 patients having follow-up and the median follow-up being only 25 months. Furthermore, no attempt was made in the present study to establish the optimum conditions for extracting the different MMPs. Finally, the ratio of MMPs to TIMPs may be more important in determining clinical outcome than levels of MMPs alone. Further studies are thus clearly necessary to establish whether MMP-1, -2, -3 or -9 are related to patient prognosis.

Irrespective of whether or not the above MMPs are related to clinical outcome, their measurement in primary cancers might be useful in predicting responses to MMP inhibitors. Inhibitors of MMPs have been shown to prevent or decrease the formation of metastases in animal model systems (Brown and Giavazzi, 1995). Furthermore, in recent years, certain MMP inhibitors have entered clinical trials. A goal to the future should be to see whether any relationship exists between the levels and the profile of MMPs in a cancer and response to this new form of cancer therapy.

### ACKNOWLEDGEMENTS

We gratefully acknowledge the excellent technical expertise of Mrs M Smet and Mr G Roland. We thank Mr H Brisy for her skilful secretarial assistance. This work was supported by the Irish Cancer Society, the International Association for Cancer Research, grants from the Communauté Française de Belgique (Actions de Recherches Concertées 93/98–171 and 95/00–191), the Commission of European Communities (Concerted European Actions BIOMED 1 no. PL931346 and BIOTECH no. CT960464), the Fonds de la Recherche Scientifique Médicale (no. 3.4573.95), the Fonds National de la Recherche Scientifique (Lotto 9.4561.94, 9.4556.95, Télévie 7.4568.95), the Association Contre le Cancer, the Association Sportive Contre le Cancer, the Centre Anticancéreux près l'Université de Liège, the CGER – Assurances 1996/1999, the Fondation Léon Frédéricq, University of Liège, the Fonds d'Investissements de Recherche Scientifique, CHU, Liège, Belgium, and the Industry Boehringer Mannheim, Penzberg, Germany. AN is a permanent research fellow from the National Fund for Scientific Research (FNRS – Brussels, Belgium). AR is a recipient of a fellowship from FNRS – Télévie.

### REFERENCES

Alvarez OA, Carmichael DF and DeClerck YA (1990) Inhibition of collagenolytic activity and metastasis of tumor cells by a recombinant human tissue inhibitor of metalloproteinase. *J Natl Cancer Inst* **82**: 588–595

Apte SS, Olsen BR and Murphy G (1995) The gene structure of tissue inhibitor of metalloproteinases (TIMP)-3 and its inhibitory activities define the distinct TIMP gene family. *J Biol Chem* **270**: 14313–14318

Atkinson SJ, Ward RV, Reynolds JJ and Murphy G (1992) Cell mediated degradation of type IV collagen and gelatin films is dependent on the activation of matrix metalloproteinases. *Biochem J* **288**: 605–611

Autio-Harmainen H, Karttunen T, Hurskainen T, Hoyhtya M, Kauppila A and Tryggvason K (1993) Expression of 72 kDa type IV collagenase (gelatinase A) in benign and malignant ovarian tumours. *Lab Invest* **69**: 312–321

© Cancer Research Campaign 1998

Aznavooran S, Murphy AN, Stetler-Stevenson WG and Liotta LA (1993) Molecular aspects of tumor cell invasion and metastasis. *Cancer* **71**: 1368–1383

Brown PD, Levy AT, Marguelies I, Liotta LA and Stetler-Stevenson WG (1990) Independent expression and cellular processing of the 72 kDa type IV collagenase and interstitial collagenase in human tumorigenic cell lines. *Cancer Res* **50**: 6184–6191

Brown PD, Bloxridge RE, Anderson E and Howell A (1993) Expression of activated gelatinase in human invasive breast carcinoma.*Clin Exp Metastasis* **11**: 183–189

Brown PD and Giavazzi R (1995) Matrix metalloproteinase inhibition: a review of anti-tumour activity. *Ann Oncol* **6**: 967–974

Chen JM, Aimes RT, Ward GR, Youndlieb GL and Quigley JP (1991) Isolation and characterisation of a 70 kDa metalloprotease (gelatinase) that is elevated in Rous sarcoma virus-transformed chicken embryo fibroblasts. *J Biol Chem* **266**: 5113–5121

Chenard MP, O'Siorain L, Shering S, Rouyer N, Lutz Y, Wolf C, Basset P, Bellocq JP and Duffy MJ (1996) High levels of stromelysin-3 correlate with poor prognosis in patients with breast carcinoma. *Int J Cancer* **69**: 448–451

Daidone MG, Silvestrini R, D'Errico A, Di Fronza G, Benini E, Mancini AM, Garbisa S, Liotta LA and Grigioni WF (1991) Laminin receptors, collagenase IV and prognosis in node-negative breast cancers. *Int J Cancer* **48**: 529–532

Davies B, Miles DW, Happerfield LC, Naylor MS, Borrow LG, Rubens RD and Balkwiller FR (1993) Activity of type IV collagenases in benign and malignant breast disease. *Br J Cancer* **67**: 1126–1131

DeClerck YA, Yean T, Ratzin BJ, Lu HS and Langley KE (1989) Purification and characterisation of two related but distinct metalloproteinase inhibitors secreted by bovine aortic endothelial cells. *J Biol Chem* **264**: 17445–17453

DeClerck YA, Perez N, Shimada H, Boone TC, Langley KC and Taylor SM (1992) Inhibition of invasion and metastasis in cells transfected with an inhibitor of metalloproteinases. *Cancer Res* **52**: 701–708.

D'Errico A, Garbisa S, Liotta LA, Castronovo V, Stetler-Stevenson WG and Grigioni WF (1991) Augmentation of collagenase IV, laminin receptor and Ki62 proliferation antigen associated with human colon, gastric and breast carcinoma progression. *Mod Pathol* **4**: 239–246

Duffy MJ (1992) The role of proteolytic enzymes in cancer invasion and metastasis. *Clin Exp Methods* **10**: 145–155

Duffy MJ, O'Siorain L, Waldron B and Smith C (1986) Estradiol receptors in human breast carcinomas assayed by use of monoclonal antibodies. *Clin Chem* **32**: 1972–1974

Duffy MJ, Blaser J, Duggan C, McDermott E, O'Higgins N, Fennelly JJ and Tschesche H (1995) Assay of matrix metalloproteinases types 8 and 9 by ELISA in human breast cancer. *Int J Cancer* **61**: 597–600

Fridman R, Fuerst TR, Bird RE, Hoyhtya M, Oelkuet M, Kraust S, Komarek D, Liotta LA, Berman ML and Stetler-Stevenson WG (1992) Domain structure of human 72 kDa gelatinase/type IV collagenase. Characterisation of proteolytic activity and identification of the tissue inhibitor of metalloproteinases-2 (TIMP-2) binding regions. *J Biol Chem* **267**: 15398–15404

Greene L, Wang M, Xiao G, Liu YE and Shi YE (1996) Loss of expression of TIMP-4, a novel human tissue inhibitor of metalloproteinase, in human breast cancer progression. *Proc Am Assoc Cancer Res* **37**: 91

Heppner KJ, Matrisian LM, Jensen RA and Rodgers WH (1996) Expression of most matrix metalloproteinase family members in breast cancer represents a tumor-induced host response. *Am J Pathol* **149**: 273–282

Heussen C and Dowdle EB (1980) Electrophoretic analysis of plasminogen activators in polyacrylamide gels containing sodium dodecyl sulfate and copolymerised substrates. *Anal Biochem* **102**: 196–202

Hoyhtya M, Hujanen E and Turpeenuemi-Hajanen T (1990) Modulation of type IV collagenase and invasive behaviour of metastatic melanoma (A 2058) cells in vitro by monoclonal antibodies to type IV collagenase. *Int J Cancer* **46**: 282–286

Ito A, Nakajima S, Sasaguri Y, Nagase H and Mori Y (1995) Co-culture of human breast adenocarcinoma MCF-7 cells and human dermal fibroblasts enhances the production of matrix metalloproteinases 1, 2 and 3 in fibroblasts. *Br J Cancer* **71**: 1039–1045

Kleiner DE and Stetler-Stevenson WG (1993) Structural biochemistry and activation of matrix metalloproteinases. *Curr Opin Cell Biol* **5**: 891–897

Leco KJ, Khokha R, Pavloff N, Hawkes SP and Edwards DR (1994) Tissue inhibitor of metalloproteinases-3 (TIMP-3) is an extracellular matrix-associated protein with a distinctive pattern of expression in mouse cells and tissues. *J Biol Chem* **269**: 9352–9360

Liabakk NB, Talbot I, Smith RA, Wilkinson K and Balkwill F (1996) Matrix metalloprotease 2 (MMP-2) and matrix metalloprotease 9 (MMP-9) type IV collagenases in colorectal cancer. *Cancer Res* **56**: 190–196

Matrisian LM (1990) Metalloproteinases and their inhibitors in matrix remodeling. *Trends Genet* **6**: 121–125

McDougall JR and Matrisian LM (1995) Contributions of tumor and stromal matrix metalloproteinases to tumour progression, invasion and metastasis. *Cancer Metastasis Rev* **14**: 351–362

Monteagudo C, Merino MJ, San-Juan J, Liotta LA and Stetler-Stevenson WG (1990) Immunohistochemical distribution of collagenase IV in normal, benign and malignant breast tissue. *Am J Pathol* **136**: 585–592

Murphy G, Nguyen Q, Cockett MI, Atkinson SJ, Allan JA, Knight CG, Willenbrock F and Docherty AJP (1994) Assessment of the role of the fibronectin-like domain of gelatinase A by analysis of a deletion mutant. *J Biol Chem* **269**: 6632–6636

Noël A, Polette M, Emonard H, Munaut C, Birembaut P and Foidart JM (1994) Coordinate enhancement of gelatinase A mRNA and activity levels in human fibroblasts in response to breast adenocarcinoma cells. *Int J Cancer* **56**: 331–336

Polette M, Clavel C, Cockett M, Giorod de Bentzmann S, Murphy G and Birembaut P (1993) Detection and localization of mRNAs encoding matrix metalloproteinases and their tissue inhibitor in human breast pathology. *Inv Metastasis* **13**: 31–37

Remacle AG, Baramova EN, Weidle UH, Krell HW and Foidart JM (1995) Purification of progelatinases A and B by continuous-elution eletrophoresis. *Prot Expr Purif* **6**: 417–422

Sato H, Takino T, Okada Y, Cao J, Shinagawa A, Yamamoto E and Seiki M (1994) A matrix metalloproteinase expressed on the surface of invasive tumour cells. *Nature* **370**: 61–64

Sier CJM, Kubben FJGM, Ganesh S, Heerding MM, Griffioen G, Hanemaaijer R, van Krieken JHJM, Lamers CBHW and Verspaget HW (1996) Tissue levels of matrix metalloproteinases MMP-2 and MMP-9 are related to the overall survival of patients with gastric carcinoma. *Br J Cancer* **74**: 413–417

Sik Lee K, Young Rha S, Joong Kim S, Hang Kim J, Kyung Roh J, Soo Kim B and Cheol Chung H (1996) Sequential activation and production of matrix metalloproteinase-2 during breast cancer progression. *Clin Exp Metastasis* **14**: 512–519

Stearns MA and Wang M (1993) Type IV collagenase (Mr 72,000) expression in human prostate: benign and malignant tissue. *Cancer Res* **53**: 878–883

Takino T, Sato H, Shinagawa A and Seiki M (1995) Identification of the second membrane-type matrix metalloproteinase (MT-MMP-2) gene from a human placenta cDNA library. *J Biol Chem* **270**: 23013–23020

Visscher DW, Hoyhtya M, Ottosen SK, Liang CM, Sarkar FH, Crissman JD and Fridman R (1994) Enhanced expression of tissue inhibitor of metalloproteinase-2 (TIMP-2) in the stroma of breast carcinomas correlates with tumour recurrence. *Int J Cancer* **59**: 339–344

Wilhelm SM, Collier IE, Marmer BL, Eisen AZ, Grant GA and Goldberg GI (1989) SV-40 transformed human lung fibroblasts secrete a 92 kDa type IV collagenase which is identical to that secreted by normal human macrophages. *J Biol Chem* **264**: 17212–17321

© Cancer Research Campaign 1998